# Eccentric Exercise for Achilles Tendinopathy: A Narrative Review and Clinical Decision-Making Considerations

**DOI:** 10.3390/jfmk4020034

**Published:** 2019-06-05

**Authors:** Dhinu J. Jayaseelan, John J. Mischke, Raymond L. Strazzulla

**Affiliations:** 1 Program in Physical Therapy, Department of Health, Human Function and Rehabilitation Sciences, The George Washington University, Washington, DC 20006, USA; 2 School of Physical Therapy and Rehabilitation Science, University of Montana, Missoula, MT 59812, USA

**Keywords:** clinical reasoning, exercise, tendinopathy

## Abstract

Background: Achilles tendinopathy is a common health condition encountered in the orthopedic and sports medicine settings. Eccentric exercise is a common intervention in the management of pain and limited function for this patient population, although contemporary evidence suggests additional exercise methods may be effective as well. Study design: Narrative review: Methods: A literature review was performed using the electronic databases Pubmed and PEDRO for articles through February 2019. Randomized clinical trials integrating eccentric exercise, with or without co-interventions, were evaluated. Outcomes related to pain and/or function were considered. A patient case is provided to highlight decision making processes related to clinical prescription of eccentrics for Achilles tendinopathy. Results: After screening titles and abstracts, seven studies were included for full review. Two articles compared eccentric exercise to a control group, four compared eccentrics to the use of modalities, while one used eccentric exercise as part of a multimodal intervention. In each case, eccentric exercise was effective in reducing pain and improving function. In comparison to other forms of exercise or additional interventions, eccentric exercise was frequently not more effective than other options. Discussion: Eccentric exercise has been associated with clinical benefit in improving pain and function for patients with Achilles tendinopathy. Despite the available evidence reporting effectiveness of eccentrics, other options may be equally useful. Appropriate load modification and exercise prescription for patients with Achilles tendinopathy requires systematic clinical reasoning and incorporation of patient values to optimize outcomes.

## 1. Background

Mid-portion Achilles tendinopathy is a common lower extremity musculoskeletal disorder with an estimated incidence of 2.16 per 1000 patient-years in the general population [1] and 10.9% in the running population [2]. If not managed properly, the injury may lead to missed athletic participation [3] and diminished quality of life [4]. Unfortunately, consensus regarding the appropriate management of AT is lacking. Recently, there has been an increased focus regarding conservative management, specifically loading parameters, for Achilles tendinopathy.

Tendons require the ability to accept, store, and deliver substantial force during everyday activities. The demand on the tendon becomes even greater during sports-related activities, where the speed and repetition of muscle activity is increased. The Achilles tendon, in particular, is commonly injured during running, when the ankle plantar flexors must alternate between various forms of muscle activity, largely eccentric. Numerous risk factors for development exist [5], and as such, the range of treatment options cover a wide spectrum to meet the individual needs of the patients. Clinical practice guidelines report strong evidence for utilizing eccentric exercise for Achilles tendinopathy [5]. As additional useful exercise prescriptions emerge, the inclusion of eccentric exercise among other forms of effective exercise in the management of tendinopathy is questioned.

Recently, a well performed systematic review using meta-analysis was completed to investigate the efficacy of heavy eccentric calf training for mid-portion Achilles tendinopathy [6]. In synthesizing results, the authors found that heavy eccentric exercise was superior to wait-and-see or modalities and soft tissue mobilization, and, also, that heavy slow resistance training may be superior to eccentric exercise. The authors noted, however, that the quality of evidence was low, and in some of the cases it was difficult to determine if the differences between groups were clinically significant. Related to the low quality of the evidence guiding exercise prescription, it is the role of the clinician to determine which, if any, exercise program or progression is most appropriate for their individual patient.

In order to make an informed decision about exercise prescription for mid-portion Achilles tendinopathy, it seems necessary to understand the quality of evidence, as well as the proposed prescription rationale and mechanism of action of eccentric exercise, current trends in management, and clinical justification for utilization in various cases. Available evidence that includes all the noted factors are currently lacking. To this end, the purpose of this article is to review the current evidence of eccentric exercise for managing Achilles tendinopathy, to discuss proposed rationales for use, and to integrate clinical decision making to assist in understanding the role of eccentric exercise in the management of Achilles tendinopathy.

## 2. Historical Perspective

Historically, one of the most well-established exercise regimens for Achilles tendinopathy is eccentric strength training. The use of eccentric training as a part of the rehabilitation of tendon injuries was established as early as 1984 by Curwin and Stanish [7]. Curwin and Stanish described a program in which the patient would perform three sets of 10 eccentric repetitions preceded and followed by static stretching. This program was performed daily and progressed in both velocity and resistance as symptoms allowed.

**Figure 1:**
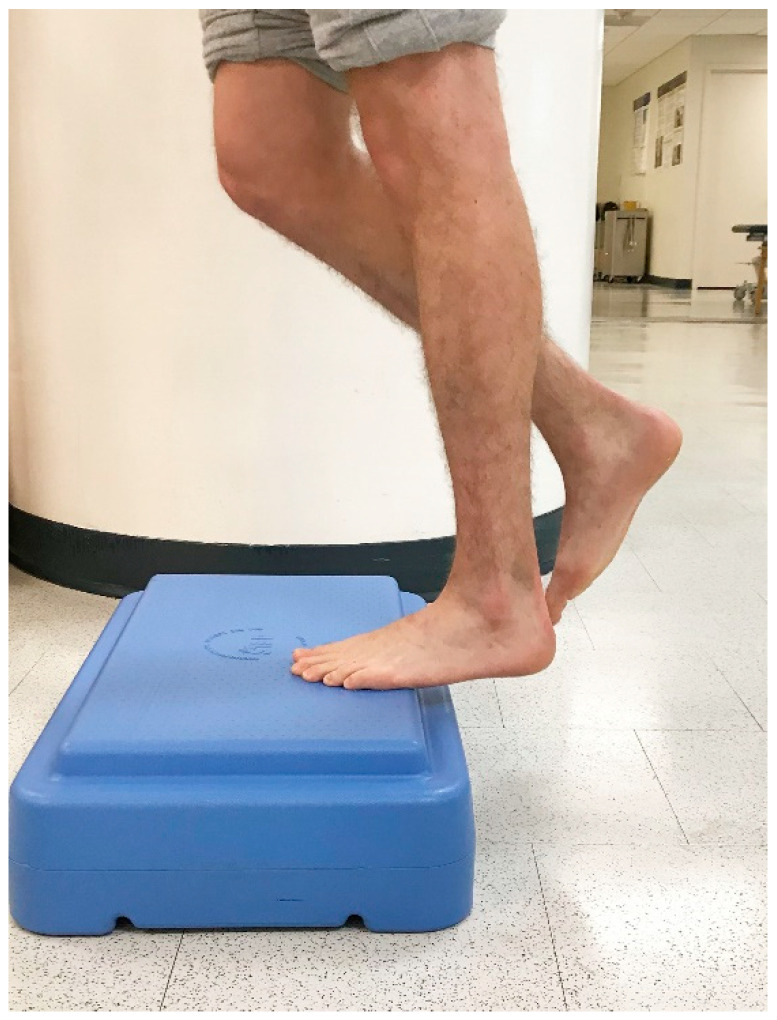
Starting position of eccentric exercise for Achilles tendinopathy. A member of the author team was used to take the photos (Figure 1, Figure 2 and Figure 3) (which do not contain any identifying information) after providing verbal consent.

**Figure 2:**
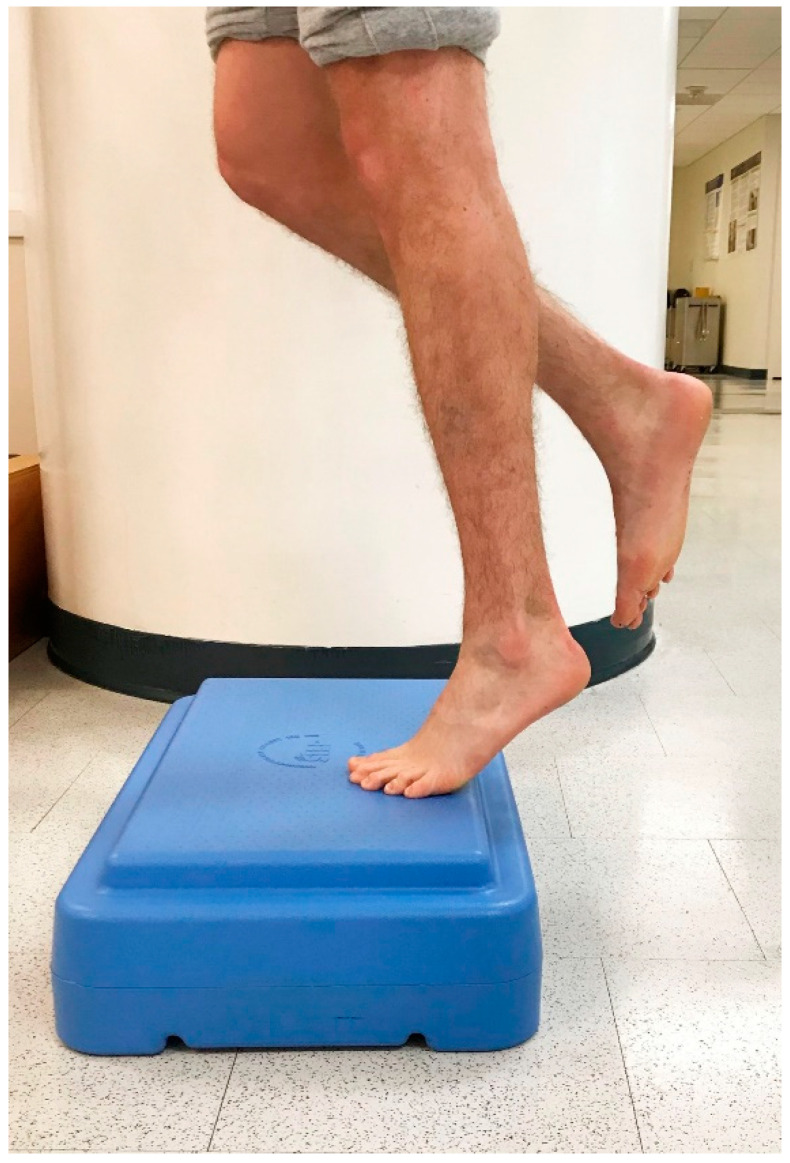
Controlled raising to end range ankle plantarflexion.

**Figure 3:**
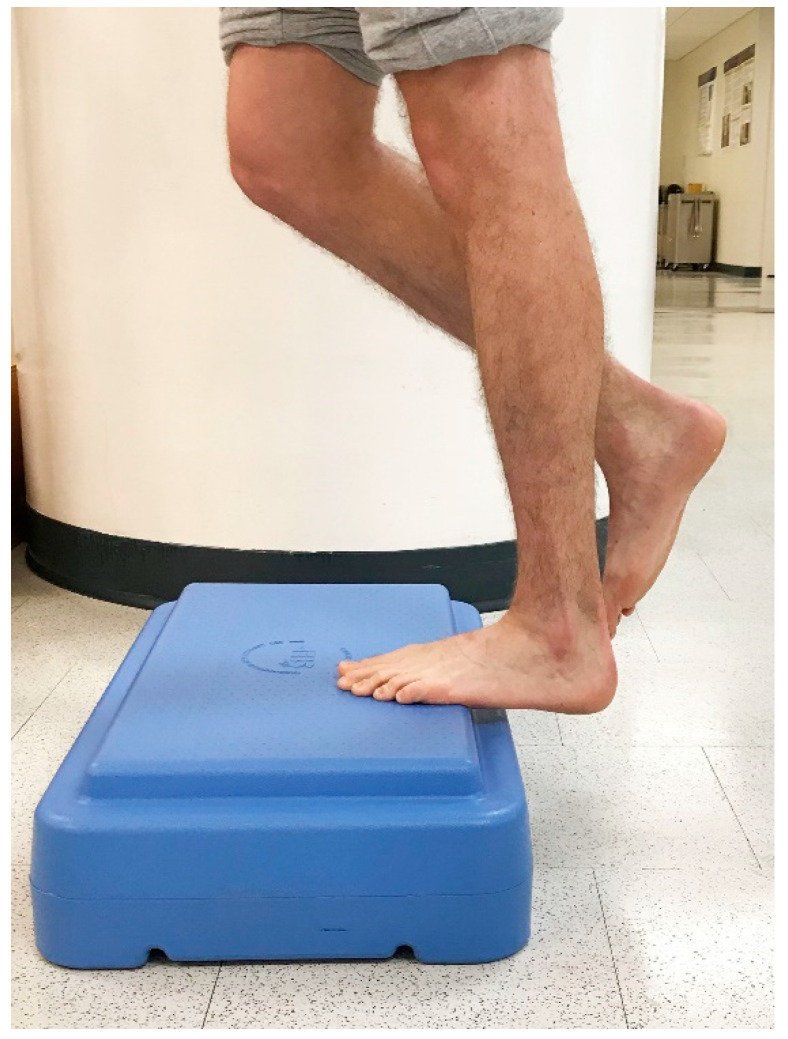
Controlled single-leg lowering into end range ankle dorsiflexion.

In 1998, Alfredson et al. modified this protocol in a prospective pilot study on recreational athletes with mid-portion Achilles tendinopathy [8]. Alfredson’s protocol consisted of a 12-week program where the subjects performed solely eccentric strengthening exercises three sets of 15 repetitions, two times daily, seven days a week. All of the 15 subjects were satisfied and returned to their prior activity level. Alfredson’s protocol suggested that the exercises be performed in the presence of pain. Eccentric loading is meant to take place into pain, and therefore resistance is added if pain is absent during the task. For this protocol, the patient started in a neutral ankle (Figure 1) position, assisted the limb into the end range of ankle plantarflexion (Figure 2), and slowly lowered the leg into the end range of ankle dorsiflexion (Figure 3). In 2000, Mafi et al. compared this same protocol to a concentric exercise regimen [9]. The eccentric group (82% satisfied) was superior to the concentric group (36% satisfied). The results of this study showed promising results for eccentric strength training; however, the results should be viewed with caution as the concentric group performed different exercises with a potential lower cumulative load as compared with the eccentric group.

In 2001, another 12-week loading program for Achilles tendinopathy was described. This program, referred to as the Silbernagel-combined protocol consisted of performing four to five exercises (concentric, eccentric, plyometric), two to three sets of 10–20 repetitions, once daily, seven days per week [10]. Follow-up studies showed promising results for this program in the treatment of Achilles tendinopathy [11,12] and a modified version of this protocol is currently being compared to the Alfredson protocol in a 52-week randomized-controlled trial [13].

Since that time, there have been numerous eccentric loading programs developed for Achilles tendinopathy, performed in isolation or with co-interventions. Many appear to be efficacious, with comparative studies appearing to show equivocal effects. However, to date there is limited evidence to suggest that using only eccentric exercises in patients with Achilles tendinopathy is more beneficial than other options [14,15]. This further emphasizes the use of clinical reasoning when prescribing exercise and loading parameters in this patient population.

## 3. Continuum Model of Tendinopathy

In 2009, Cook and Purdam proposed the load-induced tendinopathy continuum model [16]. The model sets forth three stages of tendinopathy as follows: (1) reactive tendinopathy, (2) tendon dysrepair, and (3) degenerative tendinopathy; and delineates appropriate interventions based on the stage of pathology. To briefly describe the continuum, reactive tendinopathy tends to be an acute overload more commonly seen in a younger population, whereas, degenerative tendinopathy is usually seen in the chronically overloaded tendon, more frequently noted in older individuals. The model was designed to help clinicians understand the various presentations of tendinopathy and to allow rational placement of interventions based on the continuum. The continuum model has since been revised [17], however, holds steadfast to its initial intention of altering the “one size fits all” approach and giving clinicians a platform to integrate clinical findings and critical thinking when selecting an intervention program for a patient.

## 4. Proposed Mechanisms of Action

There are currently a number of theorized mechanisms for the action of eccentric exercise in the management of Achilles tendinopathy. While the clinical efficacy is identifiable and understood, the reasons for improvement are less clear. In order to begin understanding the mechanisms of action related to eccentric exercise for Achilles tendinopathy, it may be helpful to briefly identify the alterations that occur with the clinical condition.

### 4.1. Pathological Changes with Tendinopathy

Tendon dysfunction describes a spectrum of tissue healing, and intervention prescription must match the clinical presentation to be successful. For example, with tendinitis, where clinical and/or histological signs of inflammation are present, eccentric exercise may further exacerbate the condition by not allowing for an appropriate healing process to occur. In tendinopathy however, when the true signs and symptoms of inflammation are frequently absent, eccentric exercise is more appropriate. In a degenerative tendinopathy or tendinosis, diagnostic investigation may reveal collagen disorganization, fiber separation by an increase in mucoid ground substance, increased cellular activity and vascular development, and focal necrosis or calcification [18]. Tendinopathic versus normal Achilles tendons also show a larger cross-sectional area, lower tendon stiffness, and lower Young’s modulus [19]. The result of the noted changes typically includes thickening of the tendon, which can be distinguished through palpation and a comparison to the contralateral asymptomatic limb of the patient.

While tendon degeneration is typically associated with predictable structural adaptations of enlargement, disruption of fibrillar patterns, and neovascularization [20], the reason why some individuals with tendinopathy have pain remains unclear. Some authors have suggested that pain is related to a neurovascular ingrowth into the tendon and its sheath following a failed inflammatory response to the tissue injury [21]. In persons with Achilles tendinopathy, an increased vascular flow has been noted on real-time Doppler ultrasound, as compared with individuals without the condition [22]. In a recent cross-sectional study, increased tendon thickening was associated with greater symptom severity in subjects with mid-portion Achilles tendinopathy [23]. It has also been reported that Achilles tendinopathy may be associated with altered central pain processing [24]. Conversely, a recent blinded case-control study suggests that Achilles tendinopathy is a peripherally driven pain state [25]. Given the conflicting research, more research appears to be needed to identify pain generation in Achilles tendinopathy.

### 4.2. Possible Explanations for Improvement with Eccentrics

Eccentric exercise has been shown to improve tendon structure, which historically was considered a mechanism for improvement in some persons with Achilles tendinopathy. Theoretically, if a tissue that is typically parallel in alignment (type I collagen in tendons) becomes disorganized (through tendon injury and failure of typical collagen deposition and maturation to occur in appropriate alignment) then an altered force capacity would exist, thereby limiting function. Therefore, it would make sense that normalizing the tendon structure would allow for normal tissue alignment, in turn creating a better opportunity for appropriate loading to occur. In a group of 25 patients who completed a 12-week eccentric exercise program, ultrasonographic imaging showed a localized decrease in tendon thickness and normalized tendon structure at a mean follow-up of 3.8 years [26]. The reduction in tendon thickness was correlated to patient satisfaction with treatment. The same researchers evaluated 30 patients with chronic mid-portion Achilles tendinopathy and noted a more normal tendon structure and no remaining neovascularization after 12 weeks of eccentric exercise, with an average follow-up of 28 months [27]. Again, the majority of patients who had a reduction in neovascularization reported no tendon pain during activity. In a more recent prospective cohort study of individuals with symptomatic Achilles tendinopathy, ultrasonographic tendon structure improved following eccentric loading, and normalized to values of a matched control group after 24 weeks [28]. However, a recent systematic review reported strong evidence to refute observable structural changes as an explanation for improvement of exercise for tendinopathy [29], challenging the belief that improving structure will improve function.

The lack of consistent correlation between tendon structure and symptoms [30] suggests other reasons for improvement may exist. One such rationale would be that eccentric loading more closely mimics sport-specific loading requirements than other interventions. Achilles tendinopathy is common among running sports, which requires repetitive force fluctuations through the stretch-shortening cycle (SSC), alternating between eccentric load acceptance and concentric push-off using the gastrocnemius-soleus complex. In soccer players, recurrent rates of Achilles tendinopathy, as high as 44%, have been reported [31] which may be, in part, related to an inadequate preparation before returning to sport. By increasing the strength, endurance, and power of the ankle plantar flexors through variations of eccentric exercise, participants may be more prepared for functional and sport-specific demands, and thereby reducing the rate of re-injury [32]. Some have argued that symptomatic and functional improvement for persons with tendinopathy comes with appropriate exercise prescription to the residually healthy tissue, rather than structurally modifying the abnormal tendon tissue [17]. Moreover, corticospinal changes may also exist [33], accounting for improved motor performance of previously painful tissue, which could in turn enhance sport and functional activity participation.

Eccentric exercise may be effective in treating Achilles tendinopathy in part because of its effect on pain sensitivity. In one study [34], asymptomatic individuals completed a single bout of eccentric heel raises (four sets of 15 repetitions), with pain sensitivity testing performed immediately afterwards and the next day. Pressure pain thresholds (PPT) and heat temporal summation, measures of pain sensitivity, were improved in the short term after a course of eccentric exercise. Similar effects have been seen in other body regions as well. In a sample of asymptomatic individuals, eccentric exercise (five sets of 20 repetitions) of the wrist extensors led to an immediate improvement in PPT values [35]. The exercise-induced hypoalgesic effect of eccentrics may be a component for improvement in the management of Achilles tendinopathy.

There is some evidence to suggest that tendon thickening and neovascularization may be a risk factor in developing symptomatic Achilles tendinopathy [36,37]. Hypothetically, if eccentric exercise has been shown to improve tendon structure, there may be a role for eccentric exercise in the prevention of Achilles tendinopathy. While additional theories and mechanisms for improvement may exist, the clinical benefit seen with eccentric exercise for Achilles tendinopathy may come from appropriate prescription rather than the intervention being included itself [16].

## 5. Clinical Efficacy of Eccentric Exercise for Achilles Tendinopathy

To evaluate the effectiveness of eccentric exercise for Achilles tendinopathy, a literature review was performed using the PubMed and PEDro electronic databases. Articles published through February 2019 were considered for inclusion. Additional inclusion criteria included randomized controlled trials, adults with a primary diagnosis of Achilles tendinopathy, at least one intervention group involving eccentric exercise, and primary outcomes assessing pain and/or function. Articles were excluded if they did not meet the inclusion criteria, were published greater than 15 years ago, or had no full text available in the English language. An example search string for PubMed was: (randomized clinical trial, publication type, or randomized controlled trial, publication type) and (eccentric tw, or eccentric exercise* tw, or exercise MeSH) and (tendinopathy MeSH, or Achilles tendinopathy tw, or Achilles tw, or calf tw). After filtering results based on predetermined criteria and removal of duplicates, seven articles were retained for this review. Descriptive study information of each reviewed paper is presented in Table 1.

Of the seven articles reviewed, each reported statistically significant improvements in pain for the eccentric exercise group (Table 2). Of the six articles that examined function, each reported a statistically significant improvement in function for the eccentric exercise group (Table 3). When eccentric exercise was compared to no intervention, eccentric exercise was associated with superior results. However, when eccentric exercise was compared to other interventions, modalities, or multimodal approaches, the results were mixed. Three studies showed that the integration of eccentric exercise was more effective than other forms of treatment, whereas, three articles found no statistically significant differences between intervention groups. The remaining study found a combination of eccentric exercise and laser therapy to be more effective than eccentric exercise alone [43]. None of the included studies reported other treatment options to be more effective than eccentric exercise, with or without co-interventions.

### 5.1. Eccentric Exercise Versus Control

Of the seven articles reviewed, two articles compared eccentric exercise to a control group receiving no interventions [40,42]. Both studies had similar results, with eccentric exercise groups showing significant improvements in pain and function as compared with controls. Furthermore, one study showed eccentric exercise reduced pain upon palpation, during social activities, physical activities, and while running [40].

### 5.2. Eccentric Exercise Versus other Forms of Exercise

Two articles examined a comparison between eccentric exercise and another form of exercise [38,44]. One article compared eccentric exercise to heavy slow resistance. The eccentric and heavy slow resistance groups showed statistically significant improvements for pain and function, but there were no significant between-group differences [38]. The second study compared eccentric exercise to concentric exercise [44]. This study reported that both forms of exercise led to statistically significant improvements in pain and function, although the eccentric group had greater improvements in both domains.

### 5.3. Eccentric Exercise Versus Orthoses or Therapeutic Modalities

Three articles compared eccentric exercise to various types of modalities [40,41,42]. One article compared eccentric exercise to whole body vibration, one to the AirHeel ankle orthoses, and one to shock wave therapy. Eccentric exercise was reported to have statistically significant benefits for pain upon palpation and with running as compared with whole body vibration therapy [40]. Another article reported that eccentric exercise, the AirHeel ankle orthoses, and a combination of these therapies were effective for reducing pain and improving function with no statistically significant differences between groups [41]. A third article reported that eccentric exercise and shock wave therapy were equally effective in reducing pain and improving function [42].

### 5.4. Multimodal Treatment Approaches with or Without Eccentric Exercise

One article compared a multimodal approach without eccentric exercise to eccentric exercise as part of a multimodal approach [39]. The investigators found significant improvements in pain and function when eccentric exercise was included in a protocol including deep friction massage, ultrasound, and stretching [39].

## 6. Clinical Decision-Making Considerations

On the basis of the initial studies demonstrating clinical benefit from eccentrically loading programs for Achilles tendinopathy [7,8], eccentric exercise has become somewhat of a staple in the management of the condition. According to this and previous reviews, it appears that eccentric exercise may still be effective, but other options are plausible as well [6]. With the evidence being equivocal in some instances, it falls upon the clinician to identify when, if at all, eccentric exercise should be incorporated into a patient’s treatment plan for the management of tendinopathy. The following case is presented to highlight key considerations in the decision-making process.

### 6.1. Patient Presentation

Consider a 25-year-old male who presented at physical therapy with complaints of localized right Achilles tendon pain for the previous three months. He noted his pain originally started when he began training for his first marathon. Although he was a recreational runner, he reported that he increased, somewhat rapidly, his mileage wearing new shoes. He did not have any prior history of Achilles pain, denied any parasthesias, and did not have any symptoms beyond the middle of the Achilles tendon itself. The symptoms prevented him from running any distance, walking more than 10 min without breaks, as well as going up or down stairs, without pain. The symptoms were alleviated with rest.

Upon examination, he had an antalgic gait with reduced push off on the right foot. Single-limb heel raises hurt when lifting up (the concentric phase) and were worse when coming down (the eccentric phase). Single-limb hopping was exquisitely painful on the right. Screening of the lumbar, hip, knee, and ankle joints did not recreate the primary symptoms. Neurodynamic assessment was normal. Localized tenderness of the right Achilles tendon 5 cm proximal to the calcaneus was noted.

### 6.2. Reasons for Integrating Eccentric Exercise

As previously noted, there is strong evidence that supports the use of eccentric exercise for Achilles tendinopathy. This patient appears to have Achilles tendinopathy, which is largely a clinical diagnosis. Running is a common overuse mechanism of the plantar flexor complex, and was the reported activity associated with pain in this case. He had localized tenderness to the mid-portion of the Achilles tendon, and his symptoms were made worse with tendon loading (i.e., single-leg heel raise and hopping). The absence of symptoms in additional locations makes other conditions less likely. Eccentric exercise for Achilles tendinopathy could assist in reducing the patient’s pain and facilitate a return to a running program.

### 6.3. Reasons for not Integrating Eccentric Exercise

Based on this patient’s presentation, with the absence of notable swelling, it is likely he would fit into the category of “reactive tendinopathy”. Reactive tendinopathy usually occurs after an acute overload in a younger population (in this case, a 25-year old appeared to inappropriately train for a marathon). His pain became worse with controlled and rapid use of the tendon (heel raise and hopping), and therefore it is quite likely eccentric loading would be associated with increased pain for this patient. Increasing pain and continuing to load an acutely overloaded tendon could make his condition worse. Even with his localized peripheral pain, if his pain persisted or was consistently aggravated, it is possible that sensitization of the nervous system may ensue. If nervous system sensitization or augmentation occurred, the patient’s condition may become more difficult to treat.

### 6.4. Clinical Suggestions for Incorporating Eccentric Exercise

This patient presents with a condition that would likely benefit from eccentrics, however, perhaps not currently given the high symptom irritability. The priority in this case would be to reduce the intensity and reactivity of the patient’s pain. Isometric exercise may have a hypoalgesic effect for individuals with tendinopathy [45], and it may be more appropriate in the short term. If isometrics are not effective in managing pain, lower load concentric or isotonic exercise or pain management interventions may be useful in the short term. Once pain is reduced and is more manageable, incorporation of eccentric exercise to improve tendon capacity and replicate the SSC used during functional activities such as running or jumping may be more relevant for this patient.

## 7. Discussion

Tendinopathy can be a challenging condition to manage. The overuse condition has been linked to a number of risk factors, and even after integrating evidence-supported clinical decisions, a number of individuals treated for Achilles tendinopathy develop persistent symptoms. Eccentric exercise has been used with effectiveness for the condition, and it has become almost a staple of rehabilitation programs. Contemporary evidence suggests that additional interventions may be useful as well, challenging the notion that an eccentric exercise protocol should be integrated for Achilles tendinopathy [14,15].

In this narrative review, the outcomes of seven articles using eccentric exercise for Achilles tendinopathy were evaluated. In each article, eccentric exercise was found to be efficacious, however, when compared to additional interventions, eccentric exercise did not regularly perform better. Some may evaluate the data and suggest eccentric exercise is not effective, which is quite untrue. The results of this narrative review, and previous studies, highlight that improved outcomes can be achieved using various means. A specific protocol is not necessary, rather an individualized exercise prescription to enhance tissue capacity and load management of patients with Achilles tendinopathy is more valuable.

The proposed mechanism for improvement is an ongoing point of discussion. As structural changes do not appear to be correlated to patient symptoms, a less pathoanatomical approach may be required. This is also the case, given the possible nervous system sensitization that may be associated with Achilles tendinopathy [24,46]. The consideration that exercise may enhance the tissue tolerance of healthy tissue, rather than degenerated or dysfunctional tissue, seems plausible. Achilles tendon tolerance may also be enhanced, not only by improving load capacity through exercise, but by integrating patient education, management of extrinsic risk factors (i.e., training), addressing mobility impairments as necessary, and so on.

While eccentric exercise can be useful for Achilles tendinopathy, there may be a number of reasons why it is not consistently more effective than other options. The rehabilitation of load-induced tendinopathy requires the exercise prescription to meet the demands of the patient’s functional demands and tendon capacity, which may not be met by eccentrics in each case. With the Alfredson protocol, which requires loading into pain, there is likely inherent variability between participants. Pain tolerance is subjective, and it is possible that patients using the Alfredson protocol do not appropriately load their tendon, secondary to pain. Additionally, there has been some evidence to suggest that plantaris may play a role in the development and persistence of Achilles tendinopathy [47,48,49]. Coupled with other possible local impairments [50,51], in some cases eccentric exercise may not sufficiently improve outcomes for patients with Achilles tendinopathy because it may not address the predisposing problem.

The primary limitation to our work is that this review was not intended to be systematic. Our narrative review retrieved recent articles from two electronic databases including English full-text papers only. It is possible that additional work exists on this topic. However, the presented papers and related quantitative data should provide the readers with a general representation of current evidence.

On the basis of this review, it appears that clinicians can achieve positive outcomes for mid-portion Achilles tendinopathy through the prescription of eccentric exercise, in many cases. However, given the positive outcomes related to other interventions, it is the clinician’s task to integrate the available evidence into clinical decision making in order to optimize outcomes. It is the authors’ hope that this narrative review and case discussion assists in the process.

## Figures and Tables

**Table 1:** Study descriptions.

First Author	Participants	Primary Eccentric Intervention Group	Comparison(s)	Frequency and Duration of Therapy
Beyer [38]	*n* = 47 (25 Exp, 22 Comp) Exp mean age: 48 ± 2.0; Comp mean age: 48 ± 2.0 Primary diagnosis: mid-portion Achilles tendinopathy	Unilateral eccentric exercise	Heavy slow resistance exercise	Exp: 3 × 15 repetitions per exercise; 2×/day; 7 days/week; 12 weeks Comp: 3–4 sets per exercise; 3×/week; 12 weeks total; (gradual increase in load and decrease in reps over 12 weeks)
Herrington [39]	*n* = 25 (13 Exp, 12 Comp) Exp mean age: 37 ± 9.26; Comp mean age: 36.6 ± 7.14 Primary diagnosis: Achilles tendinopathy	Eccentric exercise, DFM, US, and stretching to gastrocnemius and soleus	DFM, US, and stretching to gastrocnemius and soleus	Exp: eccentric exercise 2×/day; 7 days/week; 12 weeks total; DFM, US, and stretching followed same protocol as comp Comp: DFM and US 1×/week; 6 weeks; 6 sessions total; stretching daily; 12 weeks total
Horstmann [40]	*n* = 54 (18 Exp, 22 Comp, 14 controls) Exp mean age: 45.7 ± 8.5; Comp mean age: 46 ± 6.9; control mean age: 44.4 ± 7.7 Primary diagnosis: chronic Achilles tendinopathy	Unilateral eccentric exercise	(1) Comp: whole body vibration training (2) control	Exp: 3 × 15 repetitions per exercise; 12 weeks total; 36 total sessions Comp: 4–7 min per training session; 12 weeks total; 36 total session
Petersen [41]	*n* = 100 (37 Exp, 35 Comp, 28 combinations) Exp mean age: 42.1 ± 11.0; Comp mean age: 42.6 ± 10.7; combination mean age: 43 ± 12 Primary diagnosis: Chronic Achilles tendinopathy	Eccentric exercise	(1) AirHeel Brace (2) combination of experimental and comparison groups	Exp: 3 × 15 repetitions per exercise; 3×/day; 7 days/week; 12 weeks Comp: Participants instructed to wear the AirHeel brace during the daytime for 12 weeks Combination: Followed Exp and combination protocols
Rompe [42]	*n* = 75 (25 per group) Exp mean age: 48.1 ± 9.9; Comp mean age: 51.2 ± 10.3; control mean age: 46.4 ± 11.4 Primary diagnosis: Mid-portion Achilles tendinopathy	Eccentric exercise	(1) Shock wave Therapy (2) Control	Exp: 3 × 15 repetitions per exercise; 2×/day; 7 days/week; 12 weeks total Comp: 1×/week; 3 consecutive weeks; 3 sessions total
Tumilty [43]	*n* = 80 (20 per group) group 1 mean age: 47.2 ± 8.5; group 2 mean age: 46.2 ± 10.9; group 3 mean age: 47.7 ± 10.1; group 4 mean age: 48.5 ± 9.3 Primary diagnosis: Achilles tendinopathy	Exp group 1: Laser + eccentric exercise regime 1 Exp group 2: Laser + eccentric exercise regime 2	Comp group 1: Placebo + eccentric exercise regime 1 Comp group 2: Placebo + eccentric exercise regime 2	Exp group 1: laser therapy to Achilles tendon for 90 s, 2x/week, for 4 weeks; eccentric exercise 2x/day; 7 days/week; 12 weeks total Exp group 2: laser therapy to Achilles tendon for 90 s, 2×/week, for 4 weeks; eccentric exercise 2×/day; 2 days/week; 12 weeks total Comp group 1: Placebo laser therapy for 4 weeks; eccentric exercise 2×/day; 7 days/week; 12 weeks total Comp group 2: Placebo laser therapy for 4 weeks; eccentric exercise 2×/day; 2 days/week; 12 weeks total
Yu [44]	*n* = 32 (16 per group) Exp mean age: 20.14 ± 1.84; Comp mean age: 20.40 ± 1.27 Primary diagnosis: Achilles tendinopathy	Eccentric exercise	Concentric exercise	Exp: 3 × 15 repetitions per exercise; 50 min/session; 3 days/week; 8 weeks total Comp: 3 × 15 repetitions per concentric strengthening exercise; 5 × 10” hold per stretch; 50 min/session; 3 days/week; 8 weeks total

Abbreviations: Comp—comparison group, DFM—deep friction mobilization, Exp—experimental group, US—ultrasound.

**Table 2:** Effects of eccentric exercise on pain outcomes.

First Author	Outcome Timeline	Outcome(s) Utilized	Results ^†^
Beyer [38]	Baseline, after 12-week intervention, 52-week follow-up	(1) VAS	Eccentric-running: 49 ± 5.5, 20 ± 5.7, 12 ± 4.2 HSR-running: 54 ± 5.4, 17 ± 4.1, 5 ± 2.6 Eccentric-heel raises: 19 ± 5.0, 12 ± 3.6, 6 ± 2.6 HSR-heel raises: 29 ± 5.5, 7 ± 2.4, 5 ± 2.5 No statistically significant differences between groups at 52 weeks for VAS while running (*p* = 0.71) or during heel raises (*p* = 0.77).
Herrington [39]	Baseline, 4 weeks, 8 weeks, and 12 weeks	(1) VISA-A	Eccentric % change between time points: 30.4, 15.8 *, 5.6, 51.8 * (0–12 week % change) Comparison: 9.7, 12.8, 9.4, 31.9 * (0–12 week % change) The eccentric group demonstrated significantly higher (F = 5.21, *p* = 0.014) VISA-A scores than the comparison group over the 12-week period
Horstmann [40]	Baseline, after 12-week intervention	(1) VAS	Eccentric group impact of pain on household responsibilities: 13.0 ± 17.7, 5.5 ± 15.8 * Eccentric group impact of pain on recreation: 21.9 ± 23.0, 9.4 ± 16.9 * Eccentric group impact of pain on social activities: 9.4 ± 15.8, 1.0 ± 2.0 * Eccentric group impact of pain on running training: 76.3 ± 27.3, 24.7 ± 30.3 * Vibration group impact of pain on household responsibilities: 8.4 ± 19.7, 3.8 ± 6.2 Vibration group impact of pain on recreation: 27.2±27.3, 15.8 ± 21.3 * Vibration group impact of pain on social activities: 5.8 ± 12.9, 3.3 ± 7.0 * Eccentric group impact of pain on running training: 60.2 ± 35.0, 35.3 ± 34.7 * Control group impact of pain on household responsibilities: 16.0 ± 28.1, 10.2 ± 26.5 Control group impact of pain on recreation: 29.7 ± 30.0, 19.8 ± 26.2 Control group impact of pain on social activities: 7.2 ± 17.1, 9.9 ± 16.9 Control group impact of pain on running training: 63.9 ± 33.6, 51.0 ± 38.1 Eccentric exercise and vibration groups had significant improvements in outcomes, as compared to wait-and-see control. Eccentric group performed better in pain reduction than vibration group.
Petersen [41]	Baseline, 6 weeks, 12 weeks, 52-week follow-up	(1) VAS	Eccentric-ADLs: 20% reduction after 6 weeks *, 60% reduction after 12 weeks *, 30% reduction at 1 year * Eccentric-walking: 25% reduction after 6 weeks, 71% reduction after 12 weeks, 45% reduction at 1 year * Eccentric-sports activities: 51% reduction at 1 year * AirHeel brace-ADLs: 41% reduction after 6 weeks *, 35% reduction after 12 weeks, 27% reduction at 1 year * AirHeel brace-walking: 43% reduction after 6 weeks *, 50% reduction after 12 weeks, 46% reduction at 1 year * AirHeeel brace-sports activities: 47% reduction at 1 year * Combination-ADLs: 22% reduction after 6 weeks *, 56% reduction after 12 weeks *, 53% reduction at 1 year * Combination-walking: 36% reduction after 6 weeks *, 56% reduction after 12 weeks, 64% reduction at 1 year * Combination-sports activities: 74% reduction at 1 year * No significant between group differences seen at 1 year
Rompe [42]	Baseline, 4-month follow-up	(1) NRS	Eccentric group: 7.03 ± 0.8. 3.6 ± 2.3 Shockwave therapy group: 6.8 ± 0.9, 4.0 ± 2.2 Control group: 7.9 ± 0.6, 5.9 ± 1.8 No statistically significant difference between eccentric and SWT group (p = 0.494). Statistically significant differences between eccentric and control groups (*p* < 0.001) * and SWT and control groups (*p* < 0.001) *
Tumilty [43]	Baseline, 4 weeks, 12 weeks	(1) NPRS (pain)	Eccentric exercise regimen 1 + Laser: 8.3, 4.5 *, 2.9 * Eccentric exercise regimen 2 + Laser: 7.8, 2.4 *, 0.05 * Eccentric exercise regimen 1 + Placebo: 7.4, 4.9 *, 2.5 * Eccentric exercise regimen 2 + Placebo: 8.6, 3.5 *, 2.3 * Group 4 had statistically significant improvement in pain at 12-week follow-up. Otherwise, no significant changes in pain.
Yu [44]	Baseline, after 8-week intervention	(1) VAS	Eccentric: 5.72 ± 0.89, 2.16 ± 0.42 * Concentric: 5.72 ± 0.79, 3.26 ± 0.78 * Statistically significant changes between eccentric and concentric group in favor of eccentric

^†^ Results presented as reported data at each measurement time point. * Denotes statistically significant change. Abbreviations: ADLs—activities of daily living, NRS—numeric rating scale, NPRS—numeric pain rating scale, VAS—visual analog scale.

**Table 3:** Effects of eccentric exercise on functional outcomes.

First Author	Outcome Timeline	Outcome(s) Utilized	Results ^†^
Beyer [38]	Baseline, after 12-week intervention, 52-week follow-up	(1) VISA-A	Eccentric: 58 ± 3.9, 72 ± 3.7, 84 ± 3.5 HSR: 54 ± 3.2, 76 ± 3.7, 89 ± 2.8 No statistically significant differences between groups at 52 weeks (*p* = 0.62).
Herrington [39]	Baseline, 4 weeks, 8 weeks, and 12 weeks	(2) VISA-A	Eccentric % change between time points: 30.4 *, 15.8 *, 5.6, 51.8 * (0–12 week % change) Comparison: 9.7, 12.8, 9.4, 31.9 * (0–12 week % change) The eccentric group demonstrated significantly higher (F = 5.21, *p* = 0.014) VISA-A scores than the comparison group over the 12-week period
Petersen [41]	Baseline, 6 weeks, 12 weeks, 52-week follow-up	(1) AOFAS (2) SF-36	Eccentric AOFAS: 10% improvement at 1 year * Eccentric SF-36: 76.1 ± 21.6, 85.0 ± 16.4, 87.7 ± 12.2 * AirHeel brace AOFAS: 10% improvement at 1 year * AirHeel brace SF-36: 76.3 ± 19.8, 84.2 ± 14.4, 88.0 ± 13.0 * Combination AOFAS: 12% improvement at 1 year * Combination SF-36: 71.8 ± 24.0, 83.6 ± 21.8, 89.3 ± 17.9 * No statistically significant differences between groups
Rompe [42]	Baseline, 4-month follow-up	(1) VISA-A	Eccentric group: 50.6 ± 11.5, 75.6 ± 18.7 Shockwave therapy group: 50.3 ± 11.7, 70.4 ± 16.3 Control group: 48.2 ± 9.0, 55.0 ± 12.9 No statistically significant difference between eccentric and SWT group (*p* = 0.259). Statistically significant differences between eccentric and control groups (*p* < 0.001) * and SWT and control groups (*p* < 0.001) *
Tumilty [43]	Baseline, 4 weeks, 12 weeks	(1) VISA-A	Eccentric exercise regimen 1 + Laser: 59.9, 77.9 *, 88.6 * Eccentric exercise regimen 2 + Laser: 57.5, 81.1 *, 99.0 * Eccentric exercise regimen 1 + Placebo: 56.7, 74.7 *, 80.4 * Eccentric exercise regimen 2 + Placebo: 56.8, 77.8 *, 87.6 * Eccentric exercise regimen 2 + laser showed statistically significant improvements as compared with all other groups at 12 weeks
Yu [44]	Baseline, after 8-week intervention	(1) Biodex total Balance index	Eccentric: 36.38 ± 8.51, 8.0 ± 5.39 * Concentric: 29.0 ± 16.02, 22.50 ± 7.52 Statistically significant changes between eccentric and concentric group in favor of eccentric

^†^ Results presented as reported data at each measurement time point. * Denotes statistically significant change. Abbreviations: AOFAS—American orthopedic foot and ankle society, HSR—heavy slow resistance, SF-36—short form health survey, SWT—shockwave therapy, VISA-A—Victorian institute of sport assessment (Achilles tendon).
